# *Rice black‐streaked dwarf virus* P10 acts as either a synergistic or antagonistic determinant during superinfection with related or unrelated virus

**DOI:** 10.1111/mpp.12782

**Published:** 2019-02-14

**Authors:** Hehong Zhang, Xiaoxiang Tan, Yuqing He, Kaili Xie, Lulu Li, Rong Wang, Gaojie Hong, Junmin Li, Jing Li, Michael Taliansky, Stuart MacFarlane, Fei Yan, Jianping Chen, Zongtao Sun

**Affiliations:** ^1^ Institute of Plant Virology Ningbo University Ningbo 315211 China; ^2^ College of Plant Protection Nanjing Agricultural University Nanjing 210095 China; ^3^ The State Key Laboratory Breeding Base for Sustainable Control of Pest and Disease, Key Laboratory of Biotechnology in Plant Protection of MOA of China and Zhejiang Province, Institute of Virology and Biotechnology Zhejiang Academy of Agricultural Sciences Hangzhou 310021 China; ^4^ College of Plant Protection Northwest Agriculture and Forestry University Yangling 712100 Shaanxi China; ^5^ The James Hutton Institute, Cell and Molecular Sciences Group Invergowrie Dundee DD2 5DA UK

**Keywords:** antagonism, coat protein, coat protein‐mediated resistance, *Rice black‐streaked dwarf virus*, *Rice stripe virus*, *Southern rice black‐streaked dwarf virus*, synergism

## Abstract

*Rice black‐streaked dwarf virus* (RBSDV), a member of the genus *Fijivirus*, is a devastating pathogen of crop plants. RBSDV S10 encodes a capsid protein (P10) that is an important component of the double‐layered particle. However, little information is available on the roles of RBSDV P10 in viral infection or in interactions with other viruses. Here, we demonstrate that the expression of P10 in plants alleviates the symptoms of both RBSDV and the closely related *Southern rice black‐streaked dwarf virus* (SRBSDV), and reduces the disease incidence, but renders the plants more susceptible to the unrelated *Rice stripe virus* (RSV). Further experiments suggest that P10‐mediated resistance to RBSDV and SRBSDV operates at the protein level, rather than the RNA level, and is not a result of post‐transcriptional gene silencing. Transcriptomic data reveal that the expression of P10 in plants significantly suppresses the expression of rice defence‐related genes, which may play important roles in resistance to RSV infection. After infection with RBSDV, plants are more resistant to subsequent challenge by SRBSDV, but more susceptible to RSV. Overall, these results indicate that P10 acts as an important effector in virus interactions.

## Introduction

*Rice black‐streaked dwarf virus* (RBSDV), belonging to the genus *Fijivirus* in the family *Reoviridae*, is transmitted to rice, maize, barley and wheat by the small brown planthopper (*Laodelphax striatellus*) in a persistent, propagative manner (Wei and Li, 2016). RBSDV was first reported in Japan (Kuribayashi and Shinkai, 1952), and has caused severe economic losses to rice and maize production in Asia. Plants infected by RBSDV display severe growth abnormalities, especially severe stunting (Fang *et al.*, 2000; Shikata and Kitagawa, 1977; Wang *et al.*, 2003). RBSDV is a double‐stranded RNA virus with 10 genome segments (S1–S10). Most segments encode one protein, although S5, S7 and S9 each have two open reading frames (Zhang *et al.*, 2001). Segments S1, S2 and S3 encode an RNA‐dependent RNA polymerase (P1), a core protein (P2) and a capping enzyme (P3), respectively. The P5‐1, P6 and P9‐1 proteins together constitute the viroplasm (Akita *et al.*, 2012; Sun L *et al.*, 2013; Wang *et al.*, 2011), which is the site of viral replication and assembly. P7‐1 is a protein that forms tubules at the plasmodesmata of plant cells (Isogai *et al.*, 1998; Sun Z *et al.*, 2013b). P7‐2 is a component of the SCF complex (Skp, Cullin, F‐box‐containing complex) (Tao *et al.*, 2017). P8 and P10 are a core capsid protein and an outer capsid protein, respectively (Liu *et al.*, 2007a, b; Sun Z *et al.*, 2013a).

Mixed infections of two related or unrelated viral pathogens in the same plant commonly occur under natural conditions (Syller, 2012). In some combinations of viruses, there is an observable influence of one virus on the replication or transmission of the second virus. In a synergistic interaction, there is a positive effect on one or both viruses, resulting in an increase in viral replication or movement in the host plant. Synergistic virus infections usually have more severe effects on crop production than do single infections with either of the individual viruses. Numerous synergistic interactions have been reported (García‐Cano *et al.*, 2006; Li *et al.*, 2014; Untiveros *et al.*, 2007). Synergistic interactions frequently involve unrelated viruses: for example, a mixed infection of *Potato virus Y* (PVY) and *Potato virus X* (PVX) results in an enhancement of disease symptoms in tobacco (Bance, 1991; González‐Jara *et al.*, 2004). Mechanisms of synergism are diverse in different host–pathogen systems. For example, PVY HC‐Pro (helper component proteinase) mediates *Cucumber mosaic virus* (CMV)–PVY synergistic interactions by suppression of post‐transcriptional gene silencing (PTGS) (Fukuzawa *et al.*, 2010). *Beet curly top virus* (BCTV) C2 protein promotes *Tomato yellow leaf curl Sardinia virus* (TYLCSV) replication by creating a suitable cell environment (Caracuel *et al.*, 2012).

Antagonistic interactions often occur between related viruses. Antagonism is also referred to as cross‐protection when deployed in agricultural applications, or as superinfection exclusion (Syller and Grupa, 2016; Ziebell and Carr, 2010). This type of interaction occurs when an initial viral infection prevents or interferes with subsequent infection by a similar virus. In the field, cross‐protection involves the pre‐inoculation of crop plants with a mild or attenuated virus strain to prevent subsequent infection by severe strains of the same virus, and has been successfully used to prevent damaging infections of *Papaya ringspot virus* (PRSV) and *Citrus tristeza virus* (Folimonova, 2013; Gonsalves, 1998). The mechanistic basis for synergistic interactions between viruses has been explored in a number of studies, but much less is known about the mechanisms involved in antagonistic interactions (Ratcliff *et al.*, 1999; Ziebell and Carr, 2009; Ziebell *et al.*, 2007). Although antiviral RNA silencing may play a vital role in cross‐protection between very closely related strains of the same virus (Ratcliff *et al.*, 1999), there have been some reports that RNA silencing is not solely responsible for the mechanism of cross‐protection. For example, the CMV mutant Fny‐CMVΔ2b, which lacks the 2b silencing suppressor protein, not only provides cross‐protection against wild‐type CMV, but also against the less closely related strain TC‐CMV (Ziebell and Carr, 2009; Ziebell *et al.*, 2007).

Here, we focus on the infection of rice by the widespread, devastating virus RBSDV. Our study looks at co‐infections of rice with RBSDV and either a related reovirus, *Southern rice black‐streaked dwarf virus* (SRBSDV), or the unrelated *Rice stripe virus* (*Phenuiviridae*, *Tenuivirus*, RSV). SRBSDV is transmitted by the white‐backed planthopper (*Sogatella furcifera*) in a persistent, circulative–propagative manner (Pu *et al.*, 2012; Zhou G *et al.*, 2013). RSV propagates in, and is transmitted by, the same small brown planthopper (*Laodelphax striatellus*) which transmits RBSDV. RBSDV, SRBSDV and RSV have all caused serious disease outbreaks recently in southeastern Asia, especially in the rice fields of China (Wei *et al.*, 2009; Zhou G *et al.*, 2013).

In the experiments described here, we demonstrate that plants infected with RBSDV, or those expressing the RBSDV P10 outer capsid protein, are less susceptible to challenge with the related SRBSDV, but more susceptible to the unrelated RSV. These results have implications for both the natural development of mixed infections and the potential use of transgenic plants as a virus control strategy in the field.

## Results

### Expression of the *P10* gene in rice affects plant growth and development

**Figure 1:**
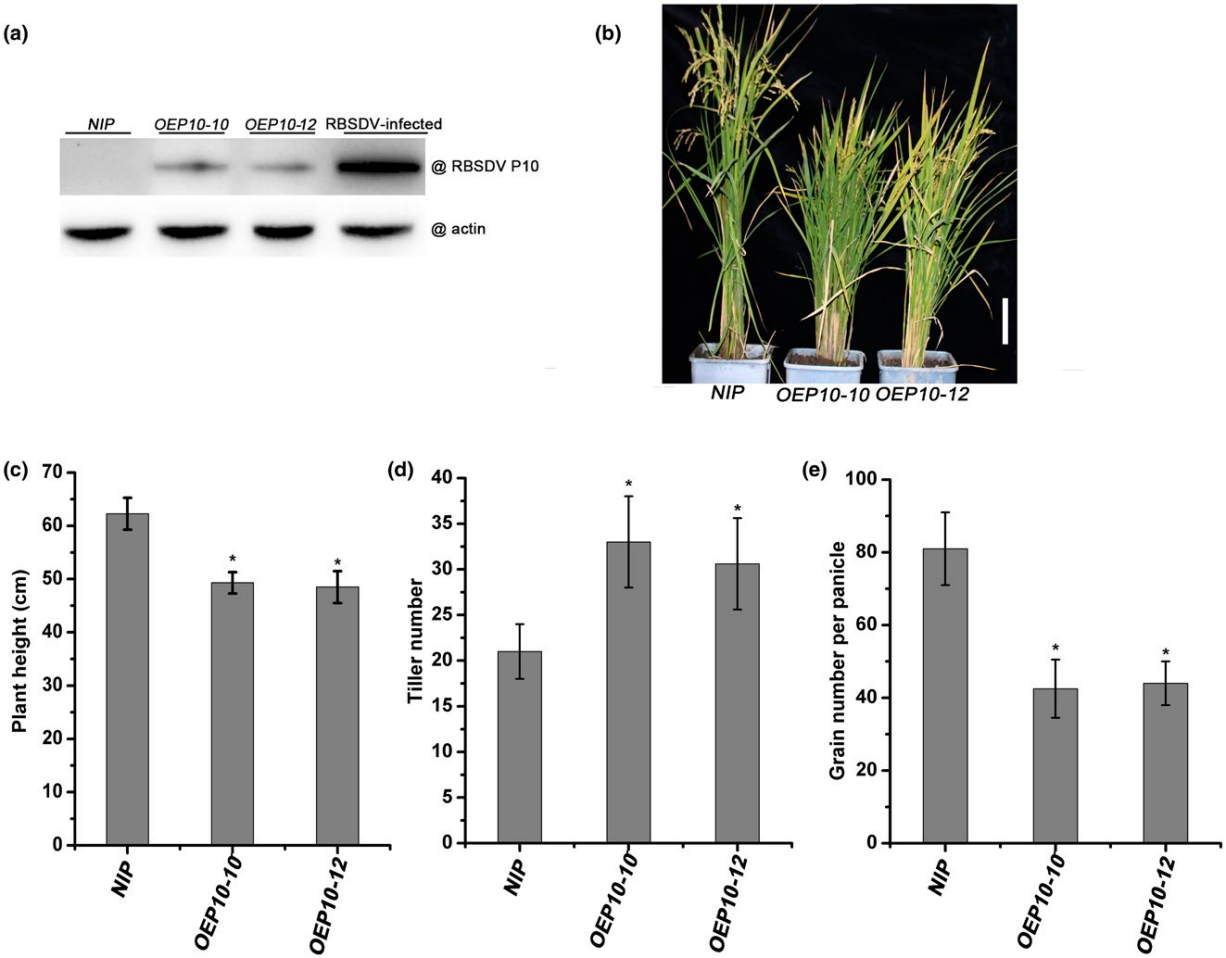
The phenotype of *OEP10 *transgenic plants expressing the *Rice black‐streaked dwarf virus* (RBSDV) P10 protein. (a) Western blotting showing P10 protein expression levels in transgenic and RBSDV‐infected plants. (b) Phenotypes of 3‐month‐old *NIP *(non‐transformed controls), *OEP10‐10 *and *OEP10‐12* plants. White bar represents 10 cm. (c) Heights of *NIP*, *OEP10‐10 *and *OEP10‐12* plants. (d) Tiller numbers in *NIP*, *OEP10‐10 *and *OEP10‐12* plants. (e) Grain numbers in each panicle of *NIP*, *OEP10‐10 *and *OEP10‐12* plants. *Significant difference at *P* < 0.05.

Our previous research has shown that RBSDV P10 is a membrane protein localized to the endoplasmic reticulum (ER) (Sun Z *et al.*, 2013a). Transient expression of P10 induces the expression of ER stress marker genes, indicating that it triggers the ER stress and unfolded protein response. In order to further study the P10 protein, we produced transgenic rice plants expressing the *P10* gene (*OEP10*) driven by the *Cauliflower mosaic virus* (CaMV) 35S promoter. Two homozygous lines and their T3 generation plants were used in subsequent experiments. The protein expression levels of P10 in *OEP10* transgenic plants were verified by western blotting (Fig. 1a). Interestingly, the phenotypes of the plants of the two transgenic lines were similar to that of the control non‐transgenic (*NIP*) plants before heading, but, at maturity, the transgenic lines were about 20% shorter than the controls (Fig. 1b,c). The transgenic plants produced many more tillers per plant (Fig. 1d) and, at the ripening stage, had a smaller number of grains per panicle (Fig. 1e). These results demonstrate that transgenic expression of the *P10* gene in rice affects both the growth and development of the plants.

### Expression of the *P10* gene in rice plants reduces their susceptibility to RBSDV

**Figure 2:**
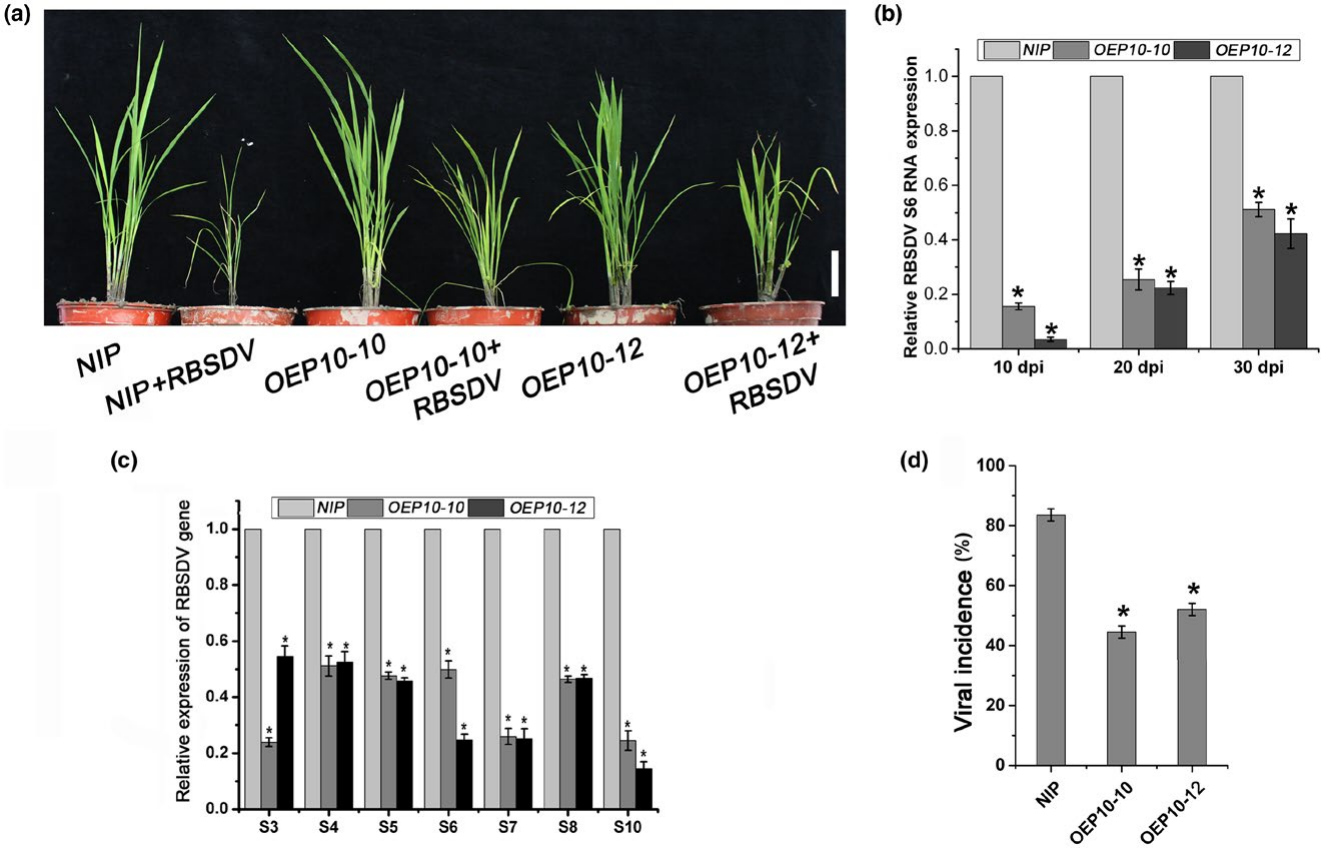
The effects of *Rice black‐streaked dwarf virus* (RBSDV) infection on *OEP10 *transgenic plants expressing the RBSDV P10 protein. (a) The visual appearance of mock‐ and RBSDV‐infected *NIP *(non‐transformed controls), *OEP10‐10 *and *OEP10‐12* plants. White bar represents 5 cm. (b) Quantitative reverse transcription‐polymerase chain reaction (RT‐qPCR) measurements of the relative expression levels of RBSDV S6 in RBSDV‐infected *OEP10‐10 *and *OEP10‐12* plants compared with that in RBSDV‐infected *NIP* at different times. (c) The relative expression levels of RBSDV genomic RNA (S3, S4, S5, S6, S7, S8, S10) levels in RBSDV‐infected *OEP10‐10 *and *OEP10‐12* plants compared with that in RBSDV‐infected *NIP* as assessed by RT‐qPCR at 30 days post‐inoculation (dpi). Relative transcript levels were analysed using the 2^–ΔΔC(t)^ method. (d) Viral incidence in *NIP*, *OEP10‐10 *and *OEP10‐12* plants. The numbers of healthy and diseased plants for each treatment were determined by RT‐PCR at 30 dpi. Each treatment used at least 30 seedlings, and three biological replicates were performed. The average values from three biological replicates are shown. Error bars represent ± standard deviation (SD). An asterisk at the top of a column indicates a significant difference at *P* < 0.05.

To further study the function of P10 in RBSDV infection, we inoculated the *OEP10* plants with RBSDV. Both transgenic lines were more resistant than control *NIP* plants to RBSDV infection. RBSDV‐infected *OEP10‐10* and *OEP10‐12* plants were less stunted than RBSDV‐infected *NIP *plants (Figs 2a and S2, see Supporting Information). In addition, we measured the viral RNA levels at different times after RBSDV inoculation. The levels of RBSDV S4, S5, S6, S7 and S10 RNA in both transgenic lines were significantly reduced compared with those in control plants at 10 days post‐inoculation (dpi), and remained at greatly reduced levels for up to 30 dpi (Figs 2b,c and S3, see Supporting Information). As shown in Fig. 2d, the viral incidence in both *OEP10‐10* and *OEP10‐12* lines (42% and 52%, respectively) was less than that in *NIP *(83%). Thus, transgenic expression of the *P10* gene in rice enhances resistance to RBSDV infection.

Previous reports have indicated that RNA‐mediated interference contributes to coat protein (CP)‐mediated viral resistance (Germundsson *et al.*, 2002; Haan *et al.*, 1992). To examine whether the P10‐mediated RBSDV resistance observed in our transgenic plants was caused by the P10 protein itself, or by the *P10* RNA, an additional transgenic rice line (*P10* ^RNA^) was generated in which the *P10* transgene was altered so that a functional P10 protein could not be expressed. Specifically, the *P10* gene was modified to insert a translation termination codon directly following the *P10* gene translation initiation codon. The morphology of *P10* ^RNA^ transgenic plants was similar to that of non‐transgenic plants. The expression level of *P10* ^RNA^ in transgenic plants was verified by western blotting. The P10 protein could not be detected in *P10* ^RNA^ plants (Fig. S1, see Supporting Information). T2 generation *P10* ^RNA^ transgenic plants were inoculated with RBSDV as described above. As shown in Fig. S4 (see Supporting Information), the viral RNA level and viral incidence in *P10* ^RNA^ transgenic plants were not significantly different from those in non‐transgenic control plants, demonstrating that it is the P10 protein, and not the *P10* RNA, that induces partial resistance to RBSDV.

**Figure 3:**
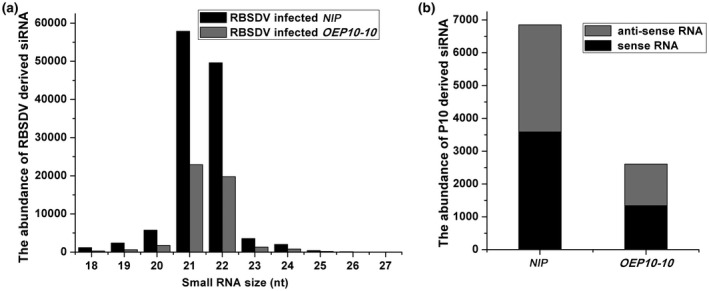
The abundance of virus‐derived small interfering RNAs (vsiRNAs) in *Rice black‐streaked dwarf virus* (RBSDV)‐infected plants. (a) The size distribution (nucleotides, nt) of RBSDV‐derived siRNAs in RBSDV‐infected plants. (b) The abundance of P10‐derived siRNAs in RBSDV‐infected plants.

Transgenic expression of the P10 protein could conceivably lead to a reduction in RBSDV infection by interfering with the small RNA‐directed anti‐virus defence response. To examine this possibility, we performed high‐throughput sequencing of small RNAs from transgenic and non‐transgenic plants inoculated with the virus. As shown in Table S2 (see Supporting Information), the size distributions of virus‐derived small interfering RNAs (vsiRNAs) in RBSDV‐infected *NIP *were mostly 21 and 22 nucleotides long, similar to previous reports (Lan *et al.*, 2017; Sun *et al.*, 2015). vsiRNAs were rarely detected in either transgenic or non‐transgenic plants (Table S2). More importantly, there were approximately 60% fewer vsiRNAs in *OEP10‐10* plants than in non‐transgenic *NIP *plants (Fig. 3 and Table S2). These results show that both the S10 RNA level (Fig. 2c) and its siRNA abundance in RBSDV‐infected *OEP10‐10* plants are lower than those in RBSDV‐infected *NIP* plants. As RNA levels and siRNA abundance are negatively correlated, we conclude that the vsiRNA‐mediated antiviral mechanism is unlikely to be responsible for the P10‐induced partial resistance to RBSDV.

### Expression of the *P10* gene in rice plants reduces their susceptibility to SRBSDV

**Figure 4:**
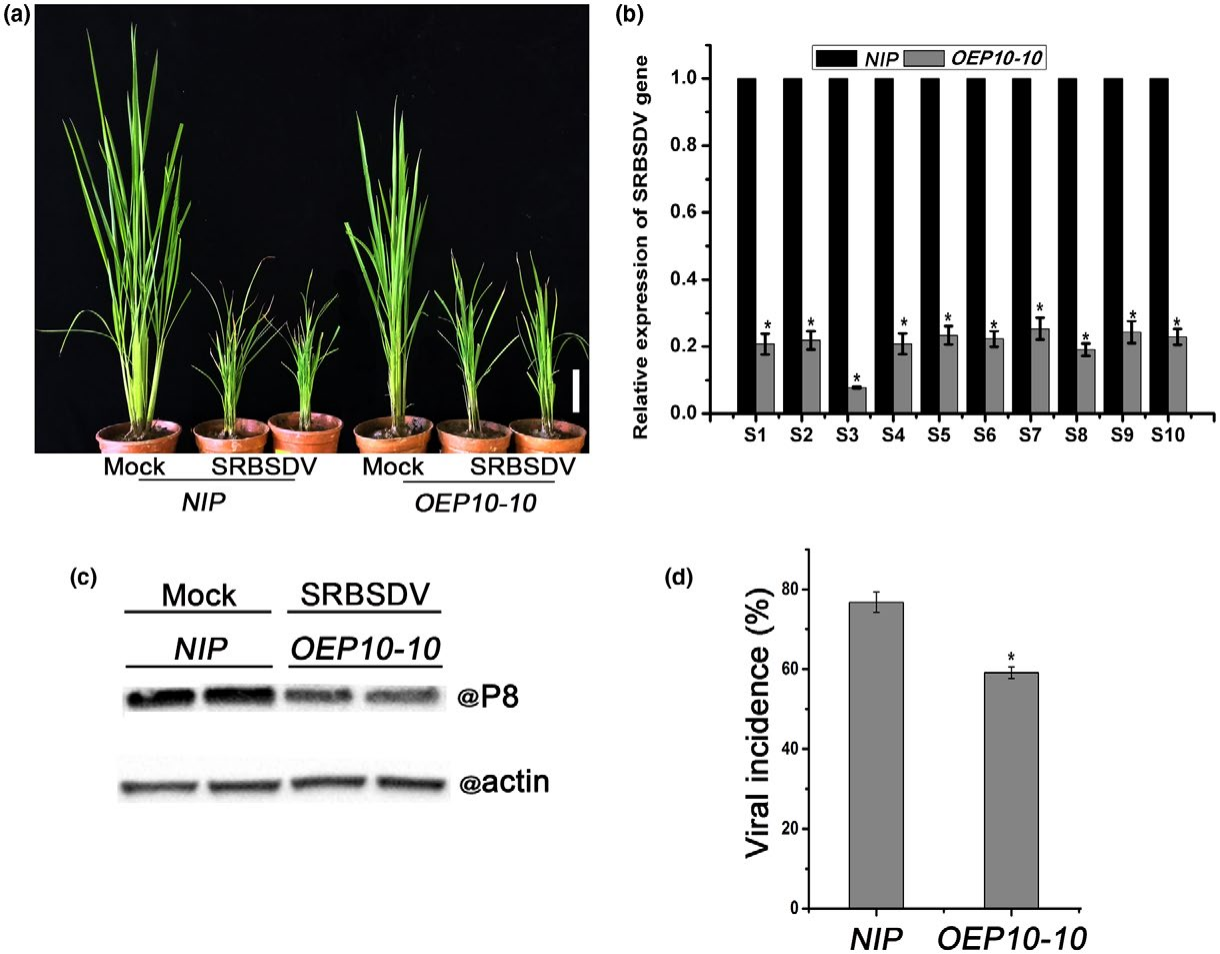
The effect of *Southern rice black‐streaked dwarf virus* (SRBSDV) infection on *OEP10 *transgenic plants expressing the *Rice black‐streaked dwarf virus* (RBSDV) P10 protein. (a) The visual appearance of mock‐ and SRBSDV‐infected plants. White bar represents 5 cm. (b) The relative expression levels of SRBSDV genes in SRBSDV‐infected plants as assessed by quantitative reverse transcription‐polymerase chain reaction (RT‐qPCR). (c) SRBSDV protein levels in SRBSDV‐infected plants determined by western blotting. (d) Viral incidence in *NIP *(non‐transformed controls) and *OEP10‐10 *plants. The numbers of healthy and diseased plants in each treatment were determined by RT‐PCR at 30 days post‐inoculation. Each treatment used at least 30 seedlings, and three biological replicates were performed. Error bars represent ± standard deviation (SD). An asterisk at the top of a column indicates significant difference at *P* < 0.05.

Because SRBSDV is a fijivirus closely related to RBSDV, we next investigated whether *OEP10* plants exhibited resistance to SRBSDV infection in the same way as to RBSDV. The transgenic *OEP10‐10 *and control *NIP* plants were infested with virus‐free and SRBSDV‐infected white‐backed planthoppers using seven insects per seedling. All planthoppers were removed after a 3‐day feeding period. As shown in Fig. 4a, SRBSDV‐infected *NIP *plants were more dwarfed and displayed more severe symptoms than did SRBSDV‐infected transgenic *OEP10‐10 *plants. Real‐time RT‐PCR showed that SRBSDV RNA levels in *OEP10‐10* plants were less than 20% of those in non‐transgenic controls (Fig. 4b). A similar difference was noted when P8 protein levels were examined by western blotting (Fig. 4c). In addition, the incidence of viral infection was significantly higher in *NIP* plants (78%) relative to *OEP10‐10* plants (60%) (Fig. 4d). As shown in Fig. S5 (see Supporting Information), the viral RNA levels and viral incidence in *OEP10‐12 *transgenic plants were similar to those in *OEP10‐10* plants in response to SRBSDV infection. These results confirm that transgenic *OEP10* plants expressing the RBSDV P10 protein show enhanced resistance to SRBSDV infection relative to *NIP* plants.

### There is mutual antagonism between RBSDV and SRBSDV in sequential infections

Because P10 enhanced the resistance of rice to both RBSDV and SRBSDV, we next tested whether there is antagonism between RBSDV and SRBSDV in sequential infections. Rice seedlings were first inoculated with RBSDV using viruliferous small brown planthoppers. Virus‐free planthoppers were used for the controls. After 7–10 days, the seedlings were inoculated with SRBSDV. Plants singly infected with RBSDV or SRBSDV were used as controls. Each treatment had two replicates each of 20–30 seedlings, and plants were then tested for viruses by RT‐PCR using primers specific for RBSDV and SRBSDV, as described previously (Cheng *et al.*, 2013). As shown in Table 1, most plants inoculated using planthoppers carrying either RBSDV or SRBSDV became infected with the respective virus. However, when insects carrying SRBSDV were fed on plants that had first been inoculated with RBSDV, most plants became infected only with RBSDV, none became infected with both RBSDV and SRBSDV, and the few plants that did develop SRBSDV infection were free from RBSDV. Similarly, in a reciprocal experiment in which plants were first inoculated with SRBSDV and then challenged with RBSDV, no plants were obtained that had a mixed infection of both viruses (Table 2). These results therefore indicate a mutual antagonism between RBSDV and SRBSDV irrespective of the order of infection.

**Table 1:** The effect of *Rice black‐streaked dwarf virus* (RBSDV) on subsequent infection by *Southern rice black‐streaked dwarf virus* (SRBSDV).

	Experiment I				Experiment II			
	Total*	RB†	SRB‡	RB‐SRB§	Total	RB	SRB	RB‐SRB
RB single inoculation	20*	20	–	–	18	15	–	–
SRB single inoculation	18	–	18	–	16	–	16	–
Primary inoculation by RB and secondary inoculation by SRB	24	24	0	0	22	18	4	0

* Total is the number of inoculated seedlings.

^†^ RB is the number of RBSDV‐infected plants.

^‡^ SRB is the number of SRBSDV‐infected plants.

^§^ RB‐SRB is the number of plants co‐infected with both RBSDV and SRBSDV.

**Table 2:** The effect of Southern rice black‐streaked dwarf virus (SRBSDV) on subsequent infection by Rice black‐streaked dwarf virus (RBSDV).

	Experiment I				Experiment II			
	Total*	SRB†	RB‡	SRB‐RB§	Total	SRB	RB	SRB‐RB
SRB single inoculation	16	10	‐	‐	20	15	‐	‐
RB single inoculation	18	‐	9	‐	20	‐	11	‐
primary inoculation by SRB and secondary inoculation by RB	18	12	1	0	25	18	2	0

* Total is the number of inoculated seedlings.

^†^ SRB is the number of SRBSDV‐infected plants.

^‡^ RB is the number of RBSDV‐infected plants.

^§^ SRB‐RB is the number of plants co‐infected with both SRBSDV and RBSDV.

### Expression of the *P10* gene in transgenic rice plants, or prior infection by RBSDV, increases susceptibility to RSV

**Figure 5:**
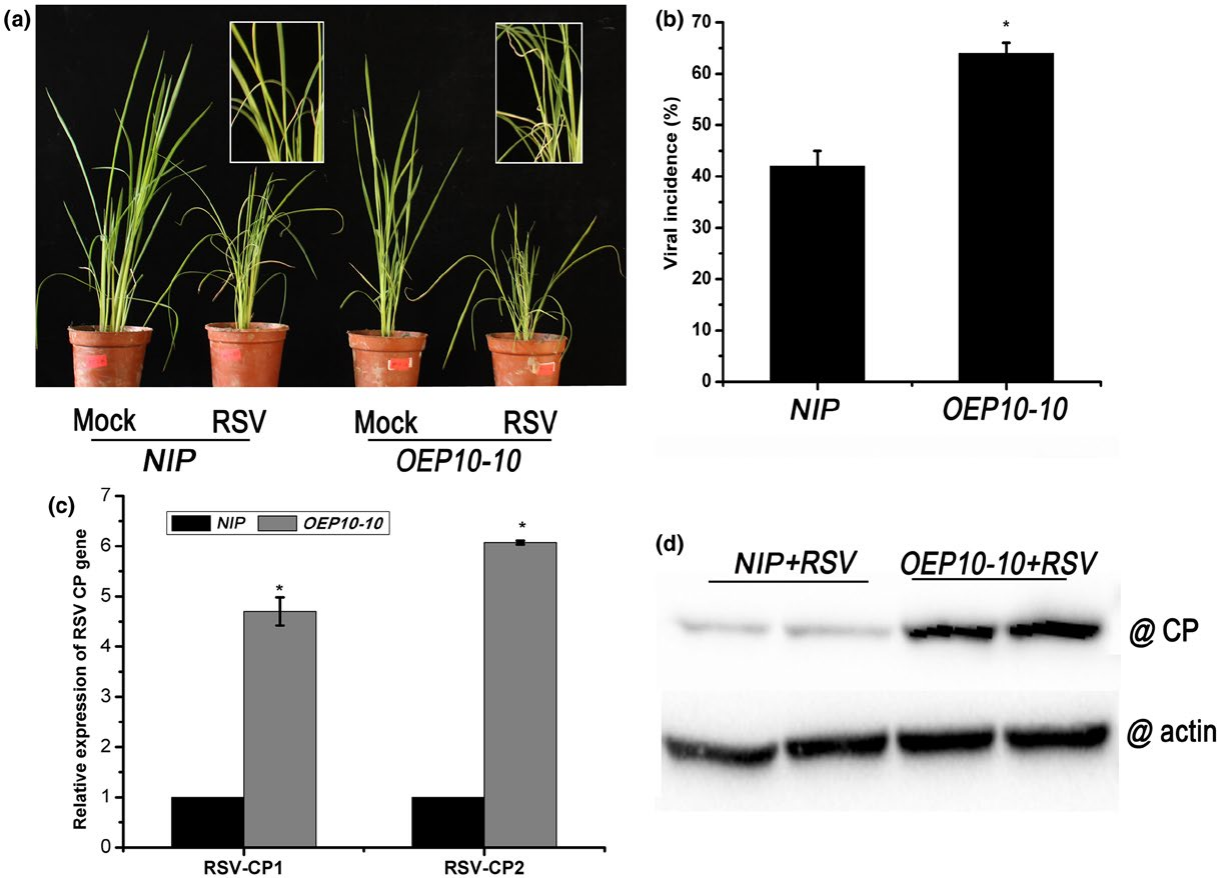
The effect of *Rice stripe virus* (RSV) infection on *OEP10 *transgenic plants expressing the *Rice black‐streaked dwarf virus* (RBSDV) P10 protein. (a) The visual appearance of mock‐ and RSV‐infected plants. (b) Viral incidence in *NIP *(non‐transformed controls) and *OEP10‐10 *plants. The numbers of healthy and diseased plants in each treatment were determined by reverse transcription‐polymerase chain reaction (RT‐PCR) at 30 days post‐inoculation. Each treatment used at least 30 seedlings, and three biological replicates were performed. (c) The relative expression levels of the RSV coat protein (CP) gene in RSV‐infected plants assessed by quantitative RT‐PCR. RSV‐CP1 and RSV‐CP2 are two pairs of primers designed at different positions in the RSV CP gene. (d) The RSV CP protein levels in RSV‐infected plants determined by western blotting. Error bars represent ± standard deviation (SD). An asterisk at the top of a column indicates a significant difference at *P* < 0.05.

To investigate how RBSDV P10 expression would affect infection by an unrelated virus, we challenged the two transgenic lines with the tenuivirus RSV using viruliferous small brown planthoppers. As shown in Fig. 5a, the symptoms in RSV‐infected control *NIP* plants were necrotic stripes, plant wilting and stunting. However, RSV‐infected *OEP10‐10 *plants showed more necrosis, increased wilting and more severe stunting. The viral incidence in *OEP10‐10 *plants (63%) was significantly greater than that in non‐transgenic *NIP* plants (41%) (Fig. 5b). Real‐time RT‐PCR measurement showed that the levels of RSV CP RNA in RSV‐infected *OEP10‐10 *plants were four‐ to five‐fold higher than those in RSV‐infected *NIP *plants (Fig. 5c). Similar results were observed for the accumulation of the RSV CP using western blotting (Fig. 5d). Similar results were obtained using transgenic *OEP10‐12* plants (Fig. S6, see Supporting Information). These results suggest that the expression of RBSDV P10 in rice plants renders them more susceptible to RSV.

**Figure 6:**
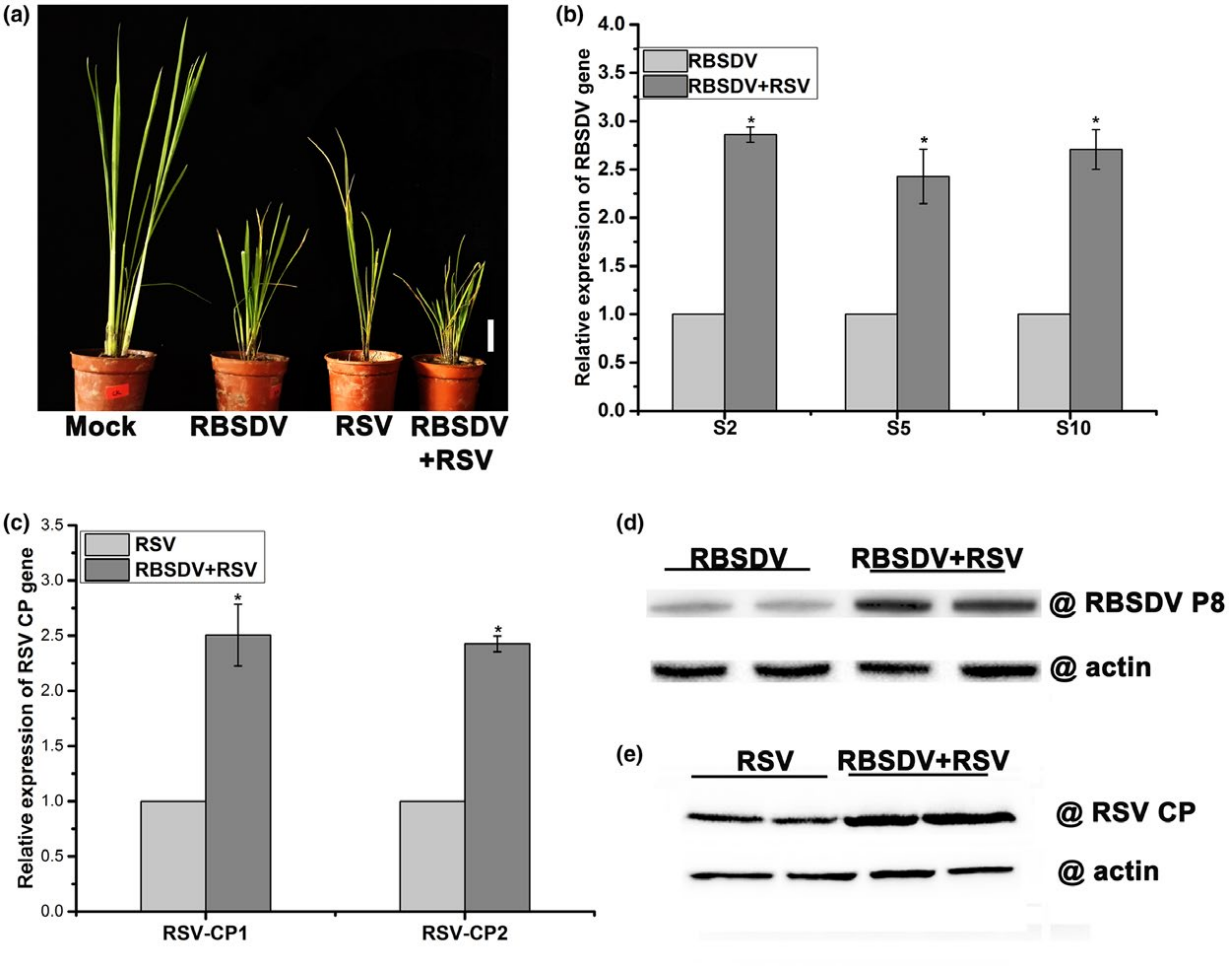
The synergistic interaction between *Rice black‐streaked dwarf virus* (RBSDV) and *Rice stripe virus* (RSV). (a) The symptoms in mock‐, RBSDV‐, RSV‐ and jointly infected plants. White bar represents 5 cm. (b) The relative expression levels of RBSDV genomic RNAs (S2, S5 and S10) in plants infected with RBSDV only or jointly with RBSDV and RSV. (c) The relative expression levels of RSV genes in plants infected with RSV only or jointly with RBSDV and RSV as shown by quantitative reverse transcription‐polymerase chain reaction (RT‐qPCR); RSV‐CP1 and RSV‐CP2 are two pairs of primers designed at different positions in the RSV coat protein (CP) gene. (d) RBSDV P8 protein levels in plants infected with RBSDV only or with RBSDV and RSV as assessed by western blotting. (e) The RSV CP protein levels in plants infected with RSV only or with both RBSDV and RSV as assessed by western blotting. Error bars represent ± standard deviation (SD). An asterisk at the top of a column indicates a significant difference at *P* < 0.05.

In natural conditions, both RBSDV and RSV are transmitted by the same vector, and natural co‐infection of rice by both viruses is common (Cho *et al.*, 2013; Li *et al.*, 2015). As P10 expression increased susceptibility to RSV, we next examined whether RBSDV itself interacts with RSV in a synergistic relationship. Rice seedlings were first inoculated with RBSDV using viruliferous small brown planthoppers and, 7 days later, these were removed and replaced with planthoppers transmitting RSV. Virus symptoms were observed at about 20 days after RSV inoculation. As shown in Fig. 6a, infection by either RBSDV or RSV alone in plants resulted in dwarfism and the development of necrotic stripes in leaves. However, more severe symptoms were observed in plants inoculated with both viruses. The relative levels of RBSDV and RSV genomic RNAs and CPs were monitored by real‐time RT‐PCR and western blotting, respectively. Consistent with the symptoms, the RBSDV and RSV RNA levels in dual‐infected rice were two‐ to three‐fold higher than those in singly infected plants (Fig. 6b,c). Similar results were observed with regard to viral protein expression levels (Fig. 6d,e). These results confirm that a synergistic interaction occurs between RBSDV and RSV, with the accumulation of both viruses being increased in the dual infection.

When RSV was inoculated first and the plants were then challenged by RBSDV, there was a different pattern of response. In the jointly infected plants, RSV titres were significantly greater than in plants infected with only RSV, but RBSDV titres were markedly lower than in those infected with only RBSDV (Fig. S8, see Supporting Information). Thus, the dual infection is always in favour of RSV, whereas the infection order determines the outcome of RBSDV multiplication.

### Transcriptome analysis of rice expressing the RBSDV P10 protein

**Figure 7:**
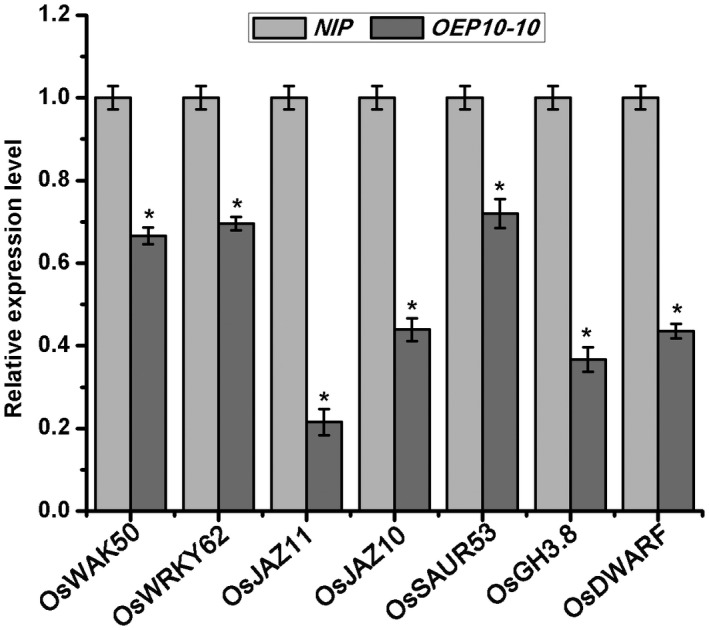
Quantitative reverse transcription‐polymerase chain reaction (RT‐qPCR) data showing the expression levels of defence response genes in *OEP10* transgenic plants expressing the *Rice black‐streaked dwarf virus* (RBSDV) P10 protein relative to those in the non‐transgenic *NIP* controls. UBQ5 was used as the internal reference gene. Data are means ± standard deviation (SD) from three biological replicates. An asterisk indicates significant difference between *NIP* and *OEP10* transgenic plants at *P* ≤ 0.05.

To shed light on the effect of expression of the RBSDV P10 protein in rice, we compared the global transcriptome of the transgenic *OEP10‐10* line with that of non‐transgenic rice plants (*NIP*). Ten‐day‐old rice seedlings were collected for RNA extraction and RNA sequencing. A total of 756 genes (523 repressed and 233 induced) were differentially expressed (two‐fold change, *P* ≤ 0.05) (Fig. S7a, see Supporting Information). A list of the genes identified is presented in Table S3 (see Supporting Information). KEGG (Kyoto Encyclopedia of Genes and Genomes) pathway analysis confirmed that P10 mainly affected plant–pathogen interaction, plant hormone signal transduction, ascorbate and aldarate metabolism and secondary metabolism, including stilbenoid biosynthesis, phenylpropanoid biosynthesis, limonene and pinene degradation and flavonoid biosynthesis pathways (Fig. S7b). For the plant–pathogen interaction pathway, the expression of 49 genes was significantly changed in the *OEP10‐10* line. The accuracy of the transcriptomic data for these defence genes was verified by quantitative reverse transcription‐polymerase chain reaction (RT‐qPCR). As shown in Fig. 7, the plant defence‐related genes, such as *WRKY* transcription factors and *JAZ *genes, were suppressed. In addition, the receptor kinase and OsWAK family belonging to the receptor signalling pathway were also altered significantly (Table 3). These results suggest that the expression of P10 in rice affects plant defence responses. In the plant hormone signalling pathways, the genes involved in auxin, cytokinin (CK) and brassinosteroid (BR) pathways were changed significantly (Table 3). These genes are involved in plant growth and development, and thus these changes are consistent with the dwarfism and developmental abnormalities seen in the P10 transgenic plants.

**Table 3:** Response of plant hormone pathway genes to expression of *P10* in rice.

Gene ID	Gene description	Log_2_(FC)*
*Plant–pathogen interaction*		
LOC_Os01g60640	WRKY21	−2.30123
LOC_Os09g25070	WRKY62	−2.02107
LOC_Os11g02520	WRKY104	−1.70669
LOC_Os05g25770	WRKY45	−1.63021
LOC_Os05g39720	WRKY70	−1.33889
LOC_Os03g53050	WRKY121	−1.32924
LOC_Os11g02480	WRKY46	−1.26024
LOC_Os09g16510	WRKY74	−1.24785
LOC_Os01g61080	WRKY24	−1.22253
LOC_Os03g28940	JAZ6	−1.10866
LOC_Os03g08320	JAZ11	−1.85962
LOC_Os10g25290	JAZ12	−1.71394
LOC_Os03g08330	JAZ10	−2.04555
*Toll‐like receptor signalling pathway*		
LOC_Os04g51040	OsWAK50	−2.09454
LOC_Os04g29580	OsWAK37	−1.54263
LOC_Os02g56370	OsWAK20	−1.47422
LOC_Os02g02120	OsWAK11	−1.43851
LOC_Os04g29960	OsWAK43	−1.40899
LOC_Os01g26280	OsWAK8	1.109275
LOC_Os04g30240	OsWAK60	1.235246
LOC_Os04g29770	OsWAK3	1.32052
LOC_Os04g21790	OsWAK34	1.544207
LOC_Os10g10130	OsWAK112d	1.54711
LOC_Os12g42070	OsWAK129b	1.722076
LOC_Os04g29680	OsWAK38	2.276714
LOC_Os09g38840	OsWAK90	5.244784
*Auxin pathway*		
LOC_Os09g37480	OsSAUR53	−3.24823
LOC_Os02g52990	OsSAUR12	−3.03287
LOC_Os07g40290	OsGH3.8	−2.20391
LOC_Os06g48950	Auxin response factor 19	−1.46253
LOC_Os01g12160	OsGH3.3	1.442901
LOC_Os09g37330	OsSAUR39	1.576482
LOC_Os01g45550	Auxin efflux carrier component	−2.07308
LOC_Os11g44810	Auxin‐repressed protein	1.880417
LOC_Os05g41420	Auxin‐induced protein 5NG4	3.915879
*Cytokinin pathway*		
LOC_Os01g56810	Cytokinin dehydrogenase	−4.2629
LOC_Os08g35860	Cytokinin dehydrogenase	1.132087
LOC_Os05g42040	UDP‐glucoronosyl and UDP‐glucosyl transferase	−1.43682
LOC_Os05g42060	UDP‐glucoronosyl and UDP‐glucosyl transferase	−1.27073
LOC_Os10g09990	Cytokinin‐*O*‐glucosyltransferase	2.581396
LOC_Os11g04720	OsRR10 type‐A response	2.618383
*Brassinosteroid pathway*		
LOC_Os03g40540	OsDWARF	−2.32283
LOC_Os07g44130	Cytochrome P450 72A1	−1.02609
LOC_Os06g02019	Cytochrome P450	1.628058
LOC_Os07g33480	Cytochrome P450	1.74535

FC, fold change.

* Transcriptomic data obtained from the comparison of *OEP10‐10 *transgenic plants expressing the *Rice black‐streaked dwarf virus* (RBSDV) P10 protein with *NIP *(non‐transformed controls).

## Discussion

RBSDV infection causes plant dwarfism and growth abnormalities, symptoms that are often associated with a change in hormone homoeostasis (He *et al.*, 2017). In a previous report, we showed that RBSDV infection affected plant hormones, including the jasmonic acid (JA) and BR pathways. In the present study, we found that expression of the RBSDV P10 protein in plants mimics these morphological changes. Transcriptome analysis indicated that expression of the P10 protein can alter the expression of genes involved in many plant biochemical pathways, including, for example, auxin, CK and BR pathways. This may be the mechanism by which infection of plants by RBSDV induces its characteristic symptoms.

In nature, the co‐infection of rice by RBSDV and SRBSDV has not been reported (Cheng *et al.*, 2013). In this study, it was not possible to superinfect RBSDV‐infected plants with SRBSDV. This phenomenon has been referred to previously as superinfection exclusion or cross‐protection (Syller and Grupa, 2016), and occurs between different strains of the same virus or sometimes between closely related viruses. Although the superinfection exclusion phenomenon has been observed with many plant‐infecting viruses (Capote *et al.*, 2006; Folimonova *et al.*, 2010; Tatineni and French, 2016; Valkonen *et al.*, 2002), only a few viral determinants involved in superinfection exclusion have been identified (Tatineni and French, 2016; Zhang *et al.*, 2017). In some studies, RNA silencing as a result of nucleotide sequence homology between pairs of viruses appears to be the mechanism underlying superinfection exclusion (Valkonen *et al.*, 2002). However, for *Wheat streak mosaic virus* (WSMV), the NIa‐Pro and CP proteins, but not their RNAs, were the determinants of superinfection exclusion (Tatineni and French, 2016). An alternative model for superinfection exclusion among plant viruses is coat protein‐mediated resistance (CPMR), a term referring to the resistance of transgenic plants expressing virus CP against infection by the same virus or similar virus strains (Beachy *et al.*, 1990; Lindbo and Falk, 2017). CPMR was first demonstrated by the resistance to *Tobacco mosaic virus* (TMV) of tobacco plants engineered to express the TMV CP gene (Abel *et al.*, 1986). Despite extensive studies, the molecular mechanism of CPMR is not fully understood, but it appears to differ depending on the virus. In the case of CPMR against TMV, the CP is known to interfere with virus particle disassembly, thereby preventing viral RNA replication (Asurmendi *et al.*, 2007; Bendahmane *et al.*, 2007). Interestingly, *OEP10* transgenic plants showed some partial resistance to SRBSDV (Fig. 4). It is possible that the P10 protein is not the sole determinant of the RBSDV–SRBSDV antagonism, or that P10 expression levels in transgenic plants were lower than those in RBSDV‐infected plants.

In our study, the expression of a wild‐type *P10 *gene in rice plants reduced their susceptibility to RBSDV and to the related virus SRBSDV. In contrast, transgenic plants expressing a mutated *P10* gene that could not be translated did not exhibit RBSDV resistance. These results suggest a P10 protein‐mediated resistance mechanism, rather than one based on nucleotide sequence homology triggering RNA silencing. Small RNA high‐throughput sequencing results suggested that siRNAs derived from the RBSDV *P10* gene were very rarely detected in *OEP10‐10* plants. The RNA levels and siRNA abundance of *P10* were both significantly reduced in RBSDV‐infected *OEP10*‐*10 *plants compared with RBSDV‐infected *NIP* controls (Fig. 3 and Table S2). These results show that the *OEP10*‐*10 *plants were partially resistant to RBSDV, but that this was not a result of the production of more *P10*‐derived or vsiRNA.

There was a synergistic interaction between the two unrelated viruses RBSDV and RSV: jointly infected plants showed an increased accumulation of RSV and of both viruses when RBSDV was inoculated first. RSV can be transmitted from female hopper adults to their progeny via eggs, whereas RBSDV does not show this transovarial transmission (Wei and Li, 2016). These two viruses cause severe yield losses and significant economic damage, and have been found in mixed infections in commercial crops (Li *et al.*, 2015). Both viruses infect and are transmitted by the same planthopper vector and, in glasshouse experiments, insects co‐infected with both viruses have been found (Li *et al.*, 2013). The synergism detected in the RBSDV–RSV superinfection, leading to an increase in both viruses in the plant, may increase the frequency/efficiency with which the insect vector can acquire each virus. This will probably increase the frequency with which the viruses are transmitted to new, uninfected plants, and so help to spread the infection of both viruses within the crop. However, a different scenario was found when RSV was infected first and RBSDV was inoculated later, suggesting that the infection order determines the outcome of the RBSDV–RSV interaction. A similar effect has been reported for PRSV and *Papaya mosaic virus* (PapMV). Synergism occurred in plants inoculated first with PRSV and later with PapMV, but there was antagonism if PapMV was inoculated first because the plant defence response was activated against PRSV (Chávez‐Calvillo *et al.*, 2016). We have not studied this, but it is possible that RSV or RSV proteins activate the plant defence response against subsequent RBSDV infection.

Viral synergistic interactions are common in mixed infections of plant viruses, and potential mechanisms behind some of these have been described (Latham and Wilson, 2008). The best‐characterized example is the interaction between PVY and PVX in tobacco plants, resulting in more severe disease symptoms (Bance, 1991). In rice, a synergistic interaction was demonstrated between SRBSDV and *Rice ragged stunt virus* (Li *et al.*, 2014): co‐infected plants showed aggravated symptoms and increased virus titres. The mechanisms of such synergism are diverse, depending on the host–pathogen systems (Latham and Wilson, 2008). For example, the RNA silencing suppressor HC‐Pro encoded by PVY mediates PVY–CMV synergistic interactions by suppression of PTGS (Fukuzawa *et al.*, 2010). The movement proteins of *Red clover mottle virus* and TMV can complement one another and promote synergism between these viruses. The *White clover yellow vein virus* (potyvirus) movement protein P3N‐PIPO facilitates the systemic spread of the *White clover mosaic virus* (potexvirus) without suppressing RNA silencing (Hisa *et al.*, 2014). The BCTV C2 protein is involved in the promotion of TYLCSV replication by creating a beneficial cell environment for viral spread (Akita *et al.*, 2012). In the present study, we found that transgenic plants expressing the RBSDV P10 protein exhibited more severe symptoms on RSV infection relative to RSV‐infected non‐transgenic control plants (Fig. 5). Transcriptome analysis and RT‐qPCR data revealed that the P10 protein affected the expression of various plant defence pathway components. KEGG pathways belonging to plant–pathogen interactions and plant hormone signal transduction networks were significantly altered. The WRKY transcription factors and JAZ proteins, which are important regulators of salicylic acid (SA) and JA signalling pathways, were significantly suppressed. Previous research has shown that SA‐mediated defence systems are involved in the suppression of RSV multiplication (Wang *et al.*, 2014). We have shown here that P10 inhibition of the plant defence response is important for both RBSDV and RSV infection and replication or movement. These results suggest that P10 is an important effector of the RBSDV–RSV synergistic interaction.

One important outcome of our work is the realization that, although transgenic approaches to virus control may have beneficial effects, as shown by the reduction in susceptibility of P10 plants to RBSDV and SRBSDV, there may also be unintended side‐effects, such as the increase in susceptibility of these plants to RSV.

## Experimental Procedures

### Plant materials and vectors

Rice cultivars *Oryza sativa* L. *japonica*. Huaidao No. 5 and Wuyujing No. 3 were used in this study. Huaidao No. 5 is highly susceptible to RBSDV, SRBSDV and RSV. Cultivar Nipponbare, which is susceptible to these viruses, was used to produce transgenic rice. RBSDV was initially collected from the field and then sequenced by our laboratory [National Center for Biotechnology Information (NCBI) reference, PRJNA14790]. RSV‐infected plants were kindly provided by Professor Yijun Zhou (Jiangsu Academy of Agricultural Sciences, China). Isolates of SRBSDV were kindly provided by Professor Guohui Zhou (South China Agricultural University, China). RBSDV and RSV were transmitted experimentally to rice plants by the small brown planthopper (*Laodelphax striatellus*), whereas SRBSDV was transmitted by the white‐backed planthopper (*Sogatella furcifera*). A small brown planthopper population was provided by the Jiangsu Academy of Agricultural Science. Viruliferous or virus‐free planthoppers were reared on healthy rice seedlings (Wuyujing No. 3) in glass beakers at 25 °C. All plants were grown in a glasshouse at 28–30 °C with a 14‐h light/10‐h dark cycle under artificial light.

### Construction of overexpression vector and rice transformation

To construct the transgenic expression vector for RBSDV P10, the RBSDV *P10* gene or the mutant of *P10* (*P10^RNA^*), carrying a translation stop codon immediately downstream of the initiation (AUG) codon, was amplified using forward and reverse primers containing *BamH*I and *Sac*I sites, respectively, as listed in Table S1 (see Supporting Information). The pCV1300 vector which contained the doubled CaMV 35S promoter to drive transcription was digested with *BamH*I and *Sac*I (Sun Z *et al.*, 2013a). The PCR product was inserted into the vector, and the selected clone was identified by sequencing. The recombinant binary plasmids were introduced into *Agrobacterium tumefaciens *(strain EHA105) using electroporation and transformed into rice cv. Nipponbare as described previously (Zhou J *et al.*, 2013).

### Virus inoculation assay

Inoculation of plants with RBSDV and RSV using small brown planthoppers was performed as described previously, with some modifications (He *et al.*, 2017; Zhou *et al.*, 2010). Three insect nymphs (either viruliferous or virus‐free) were allowed to feed on each plant for 3 days, after which the planthoppers were removed. Then, the plants were further grown in the glasshouse for symptom development to occur. The incidence of small brown planthopper infection with RBSDV or RSV was detected by a dot immunobinding assay (DIBA) (Wu *et al.*, 2013). To acquire SRBSDV, fourth‐stage nymphs of the white‐backed planthopper were fed on SRBSDV‐infected rice plants for 3–4 days. Then, three nymphs were transferred onto Wuyujing seedlings for 10 days to allow the virus to circulate through the insect. For virus‐free nymphs, insects were subjected to a similar process, but were fed on uninfected plants. Rice seedlings at the two‐ to three‐leaf stage were inoculated with three viruliferous or virus‐free nymphs per plant for 3 days and then the planthoppers were removed (Zhou G *et al.*, 2013). The infection of plants with RBSDV, SRBSDV or RSV was confirmed at 30 dpi by RT‐PCR and western blotting. Virus‐specific primers for the detection of RBSDV, SRBSDV and RSV have been described previously (Cheng *et al.*, 2013) and are listed in Table S1. The percentage of plants infected by virus (viral incidence) was determined following RT‐PCR of samples of each plant using virus‐specific primers (Table S1).

### RNA extraction and real‐time PCR

Total RNA was extracted from leaves of 30‐day‐old seedlings using TRIzol reagent (Invitrogen, Carlsbad, CA, USA) following the manufacturer’s instructions. Reverse transcription was performed using the Tiangen fast quant RT kit (Tiangen Company, Beijing, China) with 1–2 μg of total RNA in a 10‐μL reaction. RT‐qPCR assays were performed with ChamQ™ SYBR qPCR Master Mix (Low ROX Premixed), as recommended by the manufacturer, using an ABI7900HT Sequence Detection System (Applied Biosystems, Carlsbad, CA, USA). The RT‐qPCR conditions were as follows: 95 °C for 4 min; 40 cycles of 95 °C for 10 s and 60 °C for 30 s. Relative transcript levels were analysed using the 2^–ΔΔC(t) ^method (Livak and Schmittgen, 2001) by selecting rice UBQ5 as an internal control (Sun *et al.*, 2015). Each experimental result was derived from not less than three biological repeats. Each biological sample consisted of 12–15 pooled plants and was evaluated with five technical replicates. The RT‐qPCR primer sequences used in this study are listed in Table S1.

### Western blot analysis

Total protein was extracted from rice leaves with sodium dodecylsulfate (SDS) lysis buffer (100 mm Tris‐HCl, pH 6.8, 10% SDS). Then, 1 μL of 5 × SDS‐polyacrylamide gel electrophoresis (PAGE) sample loading buffer (1 m Tris‐HCl, pH 6.8, 10% SDS, 1% bromophenol blue, 50% glycerine, 2% β‐mercaptoethanol) was added to 4 μL of protein sample, and boiled at 95 °C for 10 min. The protein was separated on 10%–12% SDS‐PAGE gel and transferred to a poly(vinylidene difluoride) (PVDF) membrane. The membrane was blocked with 10% skimmed milk powder diluted in TBST (Tris‐HCl 10mm, pH = 7.4; NaCl 150mm; Tween‐20 0.05%) for 1–2 h. The primary antibody was diluted (1 : 5000) in blocking buffer (5% skimmed milk powder diluted in TBST) for 2 h. Then, secondary antibody was added in blocking buffer (1 : 10 000) and incubated according to the manufacturer’s instructions for 2 h. Antibody binding was detected using a chemiluminescent substrate (ECL; Pierce, Rockford, IL, USA). Anti‐P8 polyclonal antibody (Xie *et al.*, 2017) was used for the diagnosis of RBSDV‐infected seedlings. Anti‐RSV‐CP antibody (provided by Professor Jianxiang Wu) was used for the diagnosis of RSV‐infected rice seedlings. The actin antibody was used for the diagnosis of the reference protein (Abbkine, A01050‐3).

### Small RNA and RNA library construction and sequencing

The methods of small RNA and total RNA library construction have been described previously (He *et al.*, 2017; Sun *et al.*, 2015). The rice leaf samples were collected and ground immediately in liquid nitrogen. Total RNAs were extracted from leaves using the TRIzol protocol (Invitrogen). For RNA sequencing, the quantity and purity of total RNA, addition of adapters, size selection and RNA sequencing were performed by Zhejiang Tianke (Hangzhou, China). The total RNA sequencing library was sequenced on an Illumina HiSeq™ 2000 platform (Zhejiang Tianke Company, Hangzhou, China). The mapping of sequencing reads onto the rice genome (The MSU Rice Genome Annotation Project Database Version 7.0) was conducted using Bowtie software. The gene ontology (GO) functional classes and pathways for each sequence were determined using the Blast2go program. A difference in gene expression was considered to be significant when the absolute value of the log_2_(fold change) ratio was ≥1 and *P* ≤ 0.05.

### Statistical analysis

Differences were analysed using a one‐way analysis of variance (ANOVA) with Fisher’s least significant difference tests. *P* ≤ 0.05 was considered to be statistically significant. All analyses were performed using ORIGIN 8 software.

## Conflicts of Interest

The authors declare no competing financial interests.

## Supporting information


**Fig. S1  **Western blotting to detect P10 protein expression in *OEP10* and *P10RNA* transgenic plants.

**Fig. S2  **The height of mock‐ and *Rice black‐streaked dwarf virus* (RBSDV)‐infected (+RB) *NIP*, *OEP10‐10* and *OEP10‐12* plants. Error bars indicate ± standard deviation (SD).

**Fig. S3  **The relative expression levels of some *Rice black‐streaked dwarf virus* (RBSDV) RNAs in RBSDV‐infected *NIP*, *OEP10‐10* and *OEP10‐12* plants. RNA levels of S4, S5, S7 and S10 were tested by quantitative reverse transcription‐polymerase chain reaction (RT‐qPCR) at different times in RBSDV‐infected plants. Error bars indicate ± standard deviation (SD). An asterisk at the top of a column indicates a significant difference at *P* < 0.05.

**Fig. S4  **(A) Quantitative reverse transcription‐polymerase chain reaction (RT‐qPCR) results showing the expression levels of *Rice black‐streaked dwarf virus* (RBSDV) RNA segments S5, S6 and S8 in RBSDV‐infected *P10* ^RNA^ transgenic plants relative to the non‐transformed *NIP* controls at 30 days post‐inoculation (dpi). (B) RBSDV incidence (% plants infected) in *NIP*and *P10* ^RNA^ plants. Error bars indicate ± standard deviation (SD).

**Fig. S5  **(A) Quantitative reverse transcription‐polymerase chain reaction (RT‐qPCR) results showing the expression levels of *Southern rice black‐streaked dwarf virus* (SRBSDV) segments S1–S10 in SRBSDV‐infected *OEP10‐12 *transgenic plants relative to the non‐transformed *NIP* controls at 30 days post‐inoculation (dpi). (B) SRBSDV incidence (% plants infected) in *NIP* and *OEP10‐12* plants. Error bars indicate ± standard deviation (SD).

**Fig. S6  **(A) Quantitative reverse transcription‐polymerase chain reaction (RT‐qPCR) results showing the expression levels of the *Rice stripe virus* (RSV) coat protein gene in RSV‐infected *OEP10‐12 *transgenic plants relative to the non‐transformed *NIP* controls at 30 days post‐inoculation (dpi). Results are shown for two different primer sets (CP1 and CP2). (B) RSV incidence (% plants infected) in *NIP* and *OEP10‐12* plants. Error bars indicate ± standard deviation (SD).

**Fig. S7  **Scatterplot and KEGG (Kyoto Encyclopedia of Genes and Genomes) analysis of differential gene expression in *OEP10* transgenic plants. (A) Scatterplot analysis of differential gene expression in *OEP10‐10* plants in contrast with the non‐transformed *NIP* controls. A red dot stands for one up‐regulated gene, a green dot for one down‐regulated gene and a blue dot for one non‐significantly changed gene. Genes were considered as being expressed and differentially regulated when they complied with the following criteria: false discovery rate (FDR) < 0.05 and the absolute value of log_2_(fold change) ratio > 1. Data were taken from three biological replicates. (B) TOP 10 pathway enrichment in * OEP10‐10* plants in contrast with the controls.

**Fig. S8  **The effect of *Rice stripe virus* (RSV) on subsequent *Rice black‐streaked dwarf virus* (RBSDV) infection. (A) The relative expression levels of RBSDV genomic RNAs (S2, S5 and S10) in plants infected with RBSDV alone or jointly with RBSDV and RSV as assessed by quantitative reverse transcription‐polymerase chain reaction (RT‐qPCR) at 30 days post‐inoculation (dpi). (B) The relative expression levels of RSV coat protein (CP) gene in plants infected with RSV alone or jointly with RBSDV and RSV as assessed by RT‐qPCR at 30 dpi. Results are shown for two different primer sets (CP1 and CP2). Error bars indicate ± standard deviation (SD).

**Table S1** The primers used in this study.

**Table S2  **Overview of small RNAs from mock‐ and *Rice black‐streaked dwarf virus* (RBSDV)‐infected plants.

**Table S3** Transcriptome data obtained from *OEP10-10* and control *NIP* plants.
