# Supplementary figures and images for: Rice black‐streaked dwarf virus P10 acts as either a synergistic or antagonistic determinant during superinfection with related or unrelated virus
Source: Mol Plant Pathol. 2019 Feb 14;20(5):641–55. doi: 10.1111/mpp.12782 (PMC6637905; doi:10.1111/mpp.12782)

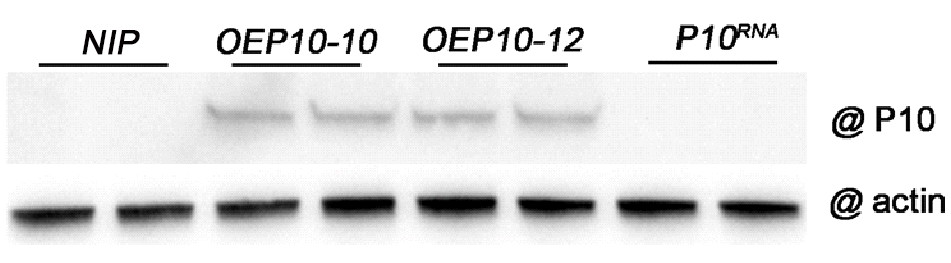


**Fig. S1.** Western blot to detect P10 protein expression in *OEP10* and *P10*RNA transgenic plants.

Supplement: Supplementary file 1 — Fig. S1 Western blotting to detect P10 protein expression in OEP10 and P10RNA transgenic plants. [file MPP-20-641-s001.docx]

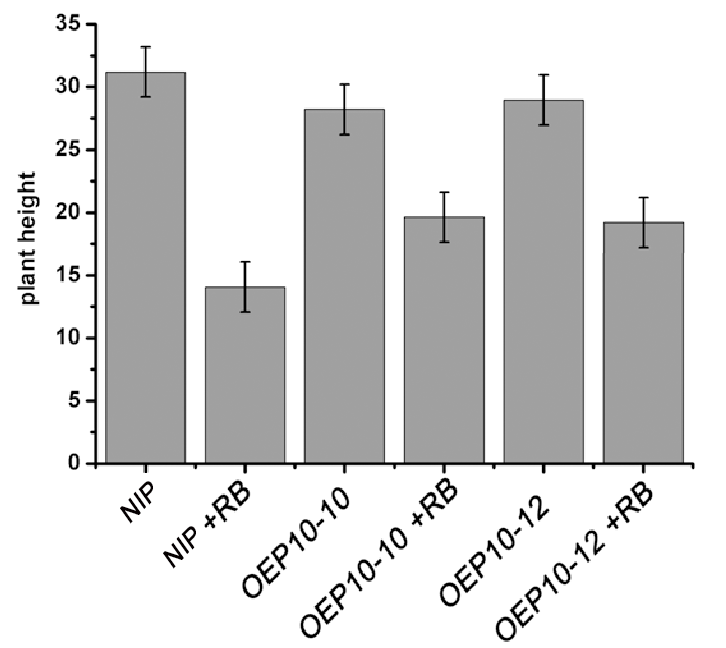


**Fig. S2.** The height of mock- or RBSDV-infected (+RB) *NIP*, *OEP10-10* and *OEP10-12* plants. Error bars indicate ±SD.

Supplement: Supplementary file 2 — Fig. S2 The height of mock‐ and Rice black‐streaked dwarf virus (RBSDV)‐infected (+RB) NIP, OEP10‐10 and OEP10‐12 plants. Error bars indicate ± standard deviation (SD). [file MPP-20-641-s002.docx]
