# Supplementary material for: Rice black‐streaked dwarf virus P10 acts as either a synergistic or antagonistic determinant during superinfection with related or unrelated virus
Source: Mol Plant Pathol. 2019 Feb 14;20(5):641–55. doi: 10.1111/mpp.12782 (PMC6637905; doi:10.1111/mpp.12782)
Supplement: Supplementary file 3 — Fig. S3 The relative expression levels of some Rice black‐streaked dwarf virus (RBSDV) RNAs in RBSDV‐infected NIP, OEP10‐10 and OEP10‐12 plants. RNA levels of S4, S5, S7 and S10 were tested by quantitative reverse transcription‐polymerase chain reaction (RT‐qPCR) at different times in RBSDV‐infected plants. Error bars indicate ± standard deviation (SD). An asterisk at the top of a column indicates a significant difference at P < 0.05. [file MPP-20-641-s003.docx]

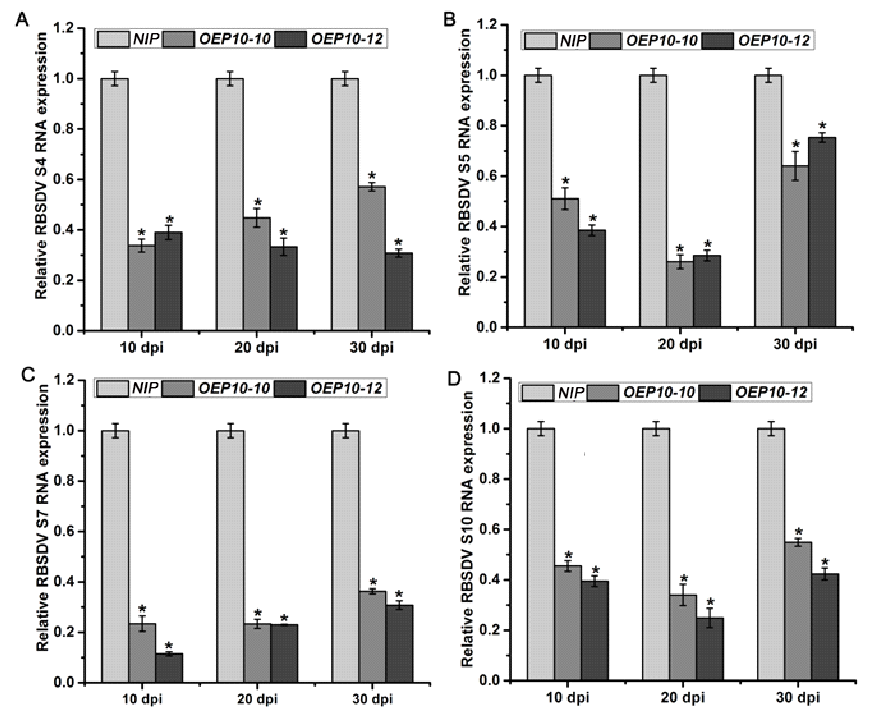


**Fig. S3.** The relative expression levels of some RBSDV RNAs in RBSDV-infected *NIP*, *OEP10-10* and *OEP10-12* plants. RNA levels of S4, S5, S7 and S10 were tested by Quantitative reverse transcription PCR (RT-qPCR) at different times in RBSDV-infected plants. Error bars indicate ±SD. * at the top of columns indicates significant difference at *p* <0.05.
