# Supplementary material for: Rice black‐streaked dwarf virus P10 acts as either a synergistic or antagonistic determinant during superinfection with related or unrelated virus
Source: Mol Plant Pathol. 2019 Feb 14;20(5):641–55. doi: 10.1111/mpp.12782 (PMC6637905; doi:10.1111/mpp.12782)
Supplement: Supplementary file 4 — Fig. S4 (A) Quantitative reverse transcription‐polymerase chain reaction (RT‐qPCR) results showing the expression levels of Rice black‐streaked dwarf virus (RBSDV) RNA segments S5, S6 and S8 in RBSDV‐infected P10 RNA transgenic plants relative to the non‐transformed NIP controls at 30 days post‐inoculation (dpi). (B) RBSDV incidence (% plants infected) in NIPand P10 RNA plants. Error bars indicate ± standard deviation (SD). [file MPP-20-641-s004.docx]

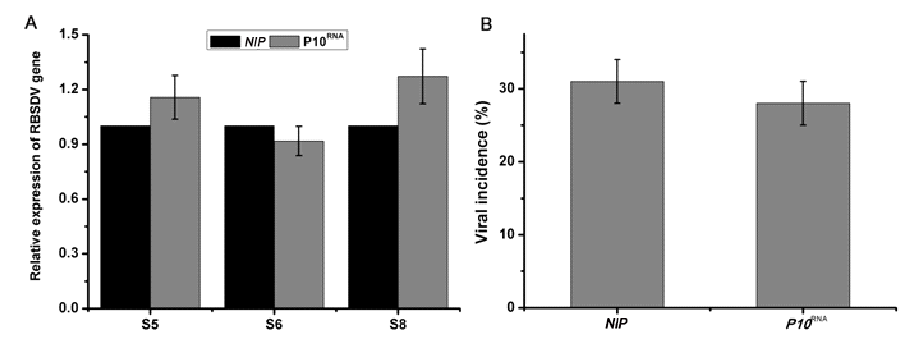


**Fig. S4.** A, RT-qPCR results showing the expression levels of RBSDV RNA segments S5, S6 and S8 in RBSDV-infected *P10*^RNA^ transgenic plants relative to the non-transformed *NIP* controls at 30 dpi. B, RBSDV incidence (% plants infected) in *NIP* and *P10*^RNA^ plants. Error bars indicate ±SD.
