# Supplementary material for: Rice black‐streaked dwarf virus P10 acts as either a synergistic or antagonistic determinant during superinfection with related or unrelated virus
Source: Mol Plant Pathol. 2019 Feb 14;20(5):641–55. doi: 10.1111/mpp.12782 (PMC6637905; doi:10.1111/mpp.12782)
Supplement: Supplementary file 5 — Fig. S5 (A) Quantitative reverse transcription‐polymerase chain reaction (RT‐qPCR) results showing the expression levels of Southern rice black‐streaked dwarf virus (SRBSDV) segments S1–S10 in SRBSDV‐infected OEP10‐12 transgenic plants relative to the non‐transformed NIP controls at 30 days post‐inoculation (dpi). (B) SRBSDV incidence (% plants infected) in NIP and OEP10‐12 plants. Error bars indicate ± standard deviation (SD). [file MPP-20-641-s005.docx]

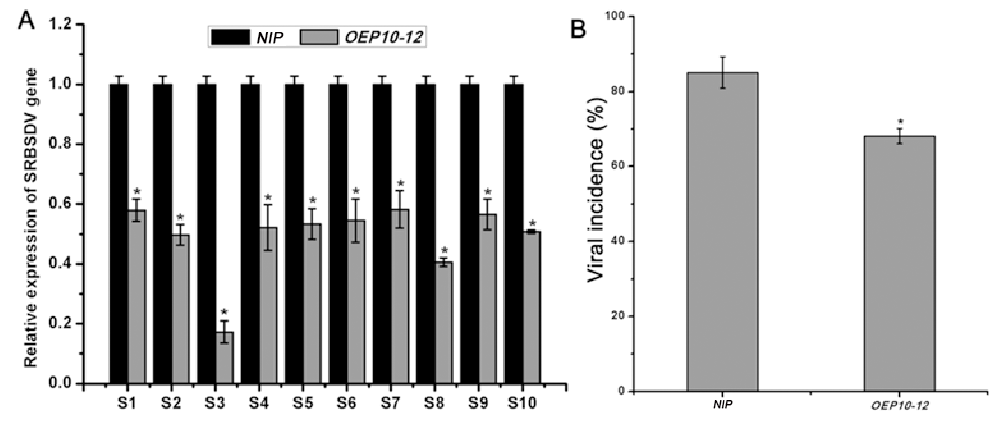


**Fig. S5.** A, RT-qPCR results showing the expression levels of SRBSDV segments S1-S10 in SRBSDV-infected *OEP10-12* transgenic plants relative to the non-transformed *NIP* controls at 30 dpi. B, SRBSDV incidence (% plants infected) in *NIP* and *OEP10-12* plants. Error bars indicate ±SD.
