# Supplementary material for: Rice black‐streaked dwarf virus P10 acts as either a synergistic or antagonistic determinant during superinfection with related or unrelated virus
Source: Mol Plant Pathol. 2019 Feb 14;20(5):641–55. doi: 10.1111/mpp.12782 (PMC6637905; doi:10.1111/mpp.12782)
Supplement: Supplementary file 6 — Fig. S6 (A) Quantitative reverse transcription‐polymerase chain reaction (RT‐qPCR) results showing the expression levels of the Rice stripe virus (RSV) coat protein gene in RSV‐infected OEP10‐12 transgenic plants relative to the non‐transformed NIP controls at 30 days post‐inoculation (dpi). Results are shown for two different primer sets (CP1 and CP2). (B) RSV incidence (% plants infected) in NIP and OEP10‐12 plants. Error bars indicate ± standard deviation (SD). [file MPP-20-641-s006.docx]

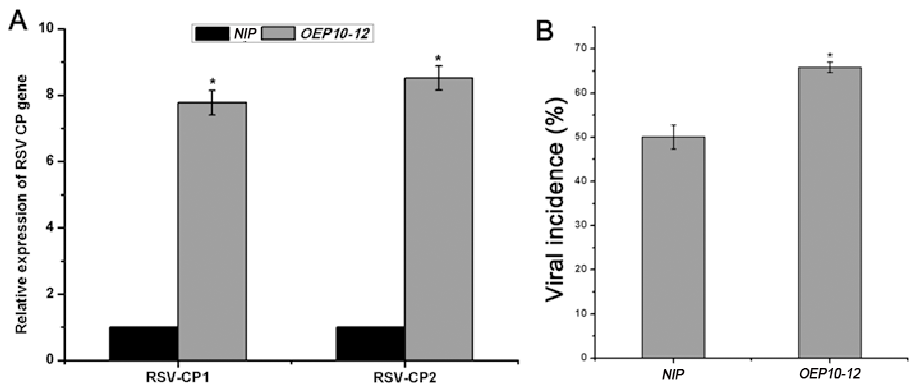


**Fig. S6.** A, RT-qPCR results showing the expression levels of the RSV coat protein gene in RSV-infected *OEP10-12* transgenic plants relative to the non-transformed *NIP* controls at 30 dpi. Results are shown for two different primer sets (CP1 and CP2). B, RSV incidence (% plants infected) in *NIP* and *OEP10-12* plants. Error bars indicate ±SD.
