# Supplementary material for: Rice black‐streaked dwarf virus P10 acts as either a synergistic or antagonistic determinant during superinfection with related or unrelated virus
Source: Mol Plant Pathol. 2019 Feb 14;20(5):641–55. doi: 10.1111/mpp.12782 (PMC6637905; doi:10.1111/mpp.12782)
Supplement: Supplementary file 7 — Fig. S7 Scatterplot and KEGG (Kyoto Encyclopedia of Genes and Genomes) analysis of differential gene expression in OEP10 transgenic plants. (A) Scatterplot analysis of differential gene expression in OEP10‐10 plants in contrast with the non‐transformed NIP controls. A red dot stands for one up‐regulated gene, a green dot for one down‐regulated gene and a blue dot for one non‐significantly changed gene. Genes were considered as being expressed and differentially regulated when they complied with the following criteria: false discovery rate (FDR) < 0.05 and the absolute value of log2(fold change) ratio > 1. Data were taken from three biological replicates. (B) TOP 10 pathway enrichment in OEP10‐10 plants in contrast with the controls. [file MPP-20-641-s007.docx]

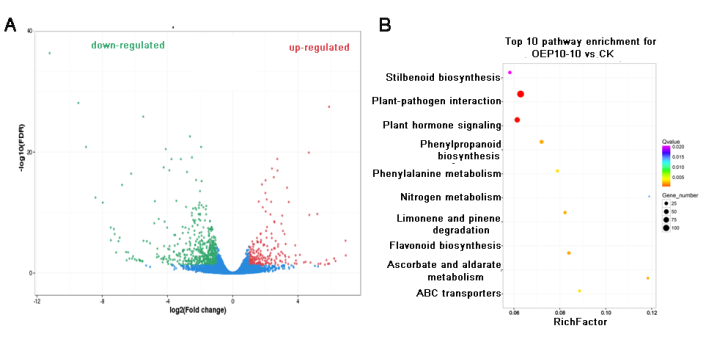


**Fig. S7.** Scatterplot and KEGG analysis of differential gene expression in *OEP10* transgenic plants. A, Scatterplot analysis of differential gene expression in *OEP10-10* plants in contrast to the non-transformed *NIP* controls. A red dot stands for one up-regulated gene, a green dot for one down-regulated gene and a blue dot stands for one non-significantly changed gene. Genes were considered as being expressed and differentially regulated when they complied with the following criteria: false discovery rate (FDR) < 0.05 and the absolute value of log_2_(fold change) ratio > 1. Data were taken from three biological replicates. B, TOP 10 pathway enrichment in *OEP10-10* plants in contrast to the controls.
