# Supplementary material for: Rice black‐streaked dwarf virus P10 acts as either a synergistic or antagonistic determinant during superinfection with related or unrelated virus
Source: Mol Plant Pathol. 2019 Feb 14;20(5):641–55. doi: 10.1111/mpp.12782 (PMC6637905; doi:10.1111/mpp.12782)
Supplement: Supplementary file 8 — Fig. S8 The effect of Rice stripe virus (RSV) on subsequent Rice black‐streaked dwarf virus (RBSDV) infection. (A) The relative expression levels of RBSDV genomic RNAs (S2, S5 and S10) in plants infected with RBSDV alone or jointly with RBSDV and RSV as assessed by quantitative reverse transcription‐polymerase chain reaction (RT‐qPCR) at 30 days post‐inoculation (dpi). (B) The relative expression levels of RSV coat protein (CP) gene in plants infected with RSV alone or jointly with RBSDV and RSV as assessed by RT‐qPCR at 30 dpi. Results are shown for two different primer sets (CP1 and CP2). Error bars indicate ± standard deviation (SD). [file MPP-20-641-s008.docx]

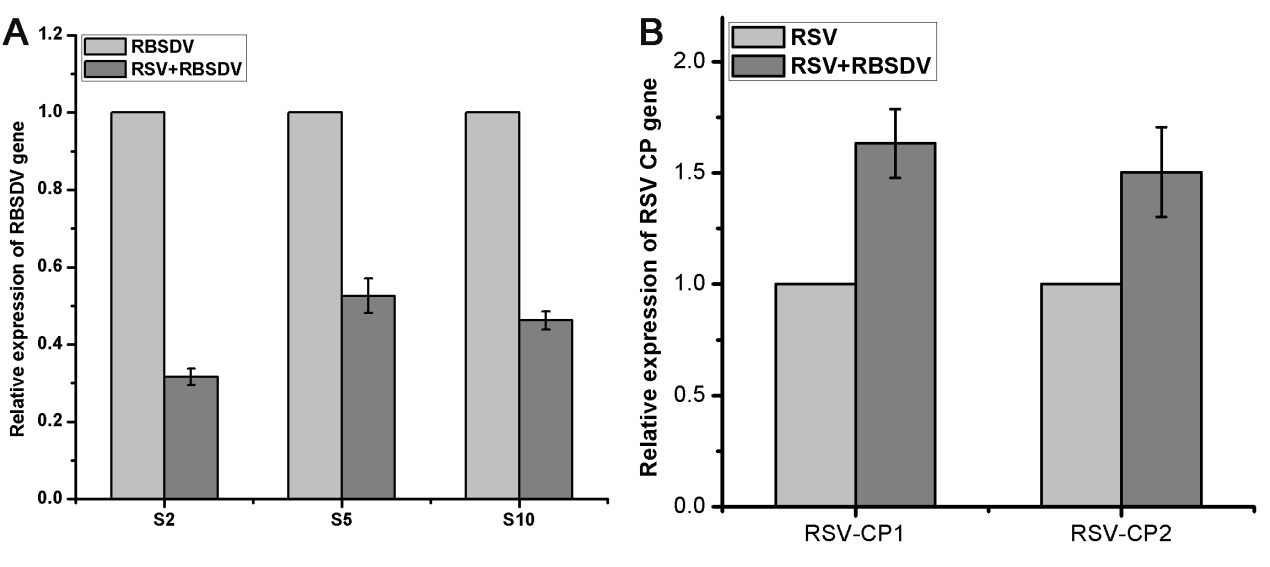


**Fig. S8.** The effect of RSV on subsequent RBSDV infection. A, The relative expression levels of RBSDV genomic RNAs (S2, S5 and S10) in plants infected with RBSDV alone or jointly with RBSDV and RSV as assessed by RT-qPCR at 30 dpi. B, The relative expression levels of RSV *CP* gene in plants infected with RSV alone or jointly with RBSDV and RSV as assessed by RT-qPCR at 30 dpi. Results are shown for two different primer sets (CP1 and CP2). Error bars indicate ±SD.
